# Supplementary material for: The Systems Biology Research Tool: evolvable open-source software
Source: BMC Syst Biol. 2008 Jun 29;2:55. doi: 10.1186/1752-0509-2-55 (PMC2446383; doi:10.1186/1752-0509-2-55)
Supplement: Additional file 1 — SBRT Archive. An archive of the current version of the Systems Biology Research Tool. [file 1752-0509-2-55-S1.zip › sbrt-1.4.0/doc/developers_guide/api/sbrt/kernel/fba/comp/FluxIntervalComputer.html]

FluxIntervalComputer


|  |  |  |
| --- | --- | --- |
| **Overview** \| **Package** \| **Class** \| **Use** \| **Tree** \| **Deprecated** \| **Index** \| **Help** | |  |
| **PREV CLASS**   **NEXT CLASS** | **FRAMES**    **NO FRAMES**     **All Classes** |  |
| SUMMARY: NESTED \| FIELD \| CONSTR \| METHOD | DETAIL: FIELD \| CONSTR \| METHOD |  |


---


## sbrt.kernel.fba.comp Interface FluxIntervalComputer

**All Superinterfaces:**: FluxCvComputer

**All Known Implementing Classes:**: AbstractFluxIntervalComputer, FastMsFluxRangeComputer, MsFluxIntervalComputer, ReverseRxnFluxIntervalComputer, SimpleFluxIntervalComputer

---

``` public interface FluxIntervalComputer extends FluxCvComputer ```

This interface is used to represent flux interval computers. Every flux in a
fluxome has a minimum and maximum value it can assume. This lower and
upper bound defines an interval. Implementors of
this interface must have the ability to compute these
flux intervals.

**Author:**
:   This interface was written and documented by Jeremiah Wright
    while in the Wagner lab.

---

| **Method Summary** | |
| --- | --- |
| `double` | `getConstraintTolerance()`             Returns the amount by which constraints can be violated. |
| `java.util.Map<java.lang.String,Interval>` | `getIntervals()`             Computes and returns the intervals of every flux in the fluxome used to construct this flux interval computer. |
| `java.util.Map<java.lang.String,Interval>` | `getIntervals(java.util.Collection<java.lang.String> rxnNames)`             Computes and returns the flux intervals of the reactions with the provided names. |
| `ProgressMonitor` | `getProgress()`             Returns a progress monitor for this flux interval computer. |
| `double` | `getZeroCutoff()`             Returns the current zero cutoff. |

**Methods inherited from interface sbrt.kernel.fba.comp.FluxCvComputer**

`getFluxConstraint, getFluxConstraints, getFluxome, getRxnExprConstraint, getRxnExprConstraints, restoreFluxConstraint, restoreFluxConstraints, restoreFluxConstraints, restoreRxnExprConstraint, restoreRxnExprConstraints, restoreRxnExprConstraints, setFluxConstraint, setFluxConstraints, setRxnExprConstraint, setRxnExprConstraints`

**Method Detail**

### getIntervals

```
java.util.Map<java.lang.String,Interval> getIntervals()
```

:   Computes and returns the intervals of every flux in the fluxome
    used to construct this flux interval computer.

    :   **Returns:**: reaction names mapped to the computed interval of their corresponding flux.

---


### getIntervals

```
java.util.Map<java.lang.String,Interval> getIntervals(java.util.Collection<java.lang.String> rxnNames)
```

:   Computes and returns the flux intervals of the reactions with the
    provided names.

    :   **Parameters:**: `rxnNames` - a collection of reaction names. **Returns:**: reaction names mapped to the computed interval of their corresponding flux.

---


### getZeroCutoff

```
double getZeroCutoff()
```

:   Returns the current zero cutoff.

    The zero cutoff is a value used to decide if coefficients are close
    enough to zero to be considered equal to zero. In other words, the
    absolute value of the coefficient of every term of this linear
    combination will be greater than the zero cutoff.

    :   **Returns:**: the zero cutoff.

---


### getConstraintTolerance

```
double getConstraintTolerance()
```

:   Returns the amount by which constraints can be violated.

    :   **Returns:**: the amount by which constraints can be violated.

---


### getProgress

```
ProgressMonitor getProgress()
```

:   Returns a progress monitor for this flux interval computer.

    :   **Returns:**: a progress monitor for this flux interval computer.


---


|  |  |  |
| --- | --- | --- |
| **Overview** \| **Package** \| **Class** \| **Use** \| **Tree** \| **Deprecated** \| **Index** \| **Help** | |  |
| **PREV CLASS**   **NEXT CLASS** | **FRAMES**    **NO FRAMES**     **All Classes** |  |
| SUMMARY: NESTED \| FIELD \| CONSTR \| METHOD | DETAIL: FIELD \| CONSTR \| METHOD |  |


---
